# Supplementary material for: Defective pgsA contributes to increased membrane fluidity and cell wall thickening in S. aureus with high-level daptomycin resistance
Source: bioRxiv. 2024 Apr 17:2023.04.11.536441. Originally published 2023 Apr 11. Preprint. [Version 3] doi: 10.1101/2023.04.11.536441 (PMC10120677; doi:10.1101/2023.04.11.536441)
Supplement: Supplement 1 [file media-1.pdf]

Supporting Information

for

Defective pgsA contributes to increased membrane fluidity and cell wall thickening in S. aureus with high-level daptomycin resistance

Christian D. Freeman[a], Tayte Hansen[b], Ramona Urbauer[a], Brian J. Wilkinson[b], Vineet K. Singh[c], and Kelly M. Hines[a #]

[a] Department of Chemistry, University of Georgia, Athens, GA, USA

[b] School of Biological Sciences, Illinois State University, Normal, IL, USA

[c] Department of Microbiology and Immunology, A.T. Still University of Health Sciences, Kirksville, MO, USA

[#]Address correspondence to: Kelly M. Hines, kelly.hines@uga.edu

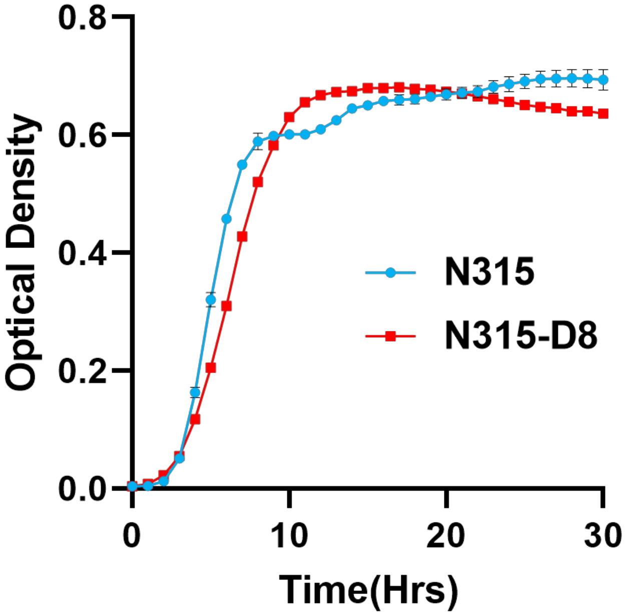

Figure S1. Growth curves for N315 and N315-D8 in TSB. Optical density was monitored at 600nm.

Table S1. Mutations detected in N315-D8 as previously reported by Hines et al., 2017.

**Predicted Gene Product**	**Nucleotide Change in N315-D8**	**Predicted Amino Acid Change in N315-D8**	**Predicted Protein Function**
yycG	1278 G → A	M426I	Fatty acid biosynthesis<br>Cell wall biosynthesis
pgsA	403 A → G	K135E	PG biosynthesis
mprF	2476 C → T	L826F	LysylPG biosynthesis
SA0567	865 C → T	Q289*	Iron complex transport
norA	737 G → C	G246A	Quinolone resistance protein
rnr	1819 G → T	E607*	Ribonuclease
spoIIIE	Deletion of 2059 C	Q687 frameshift	DNA translocase

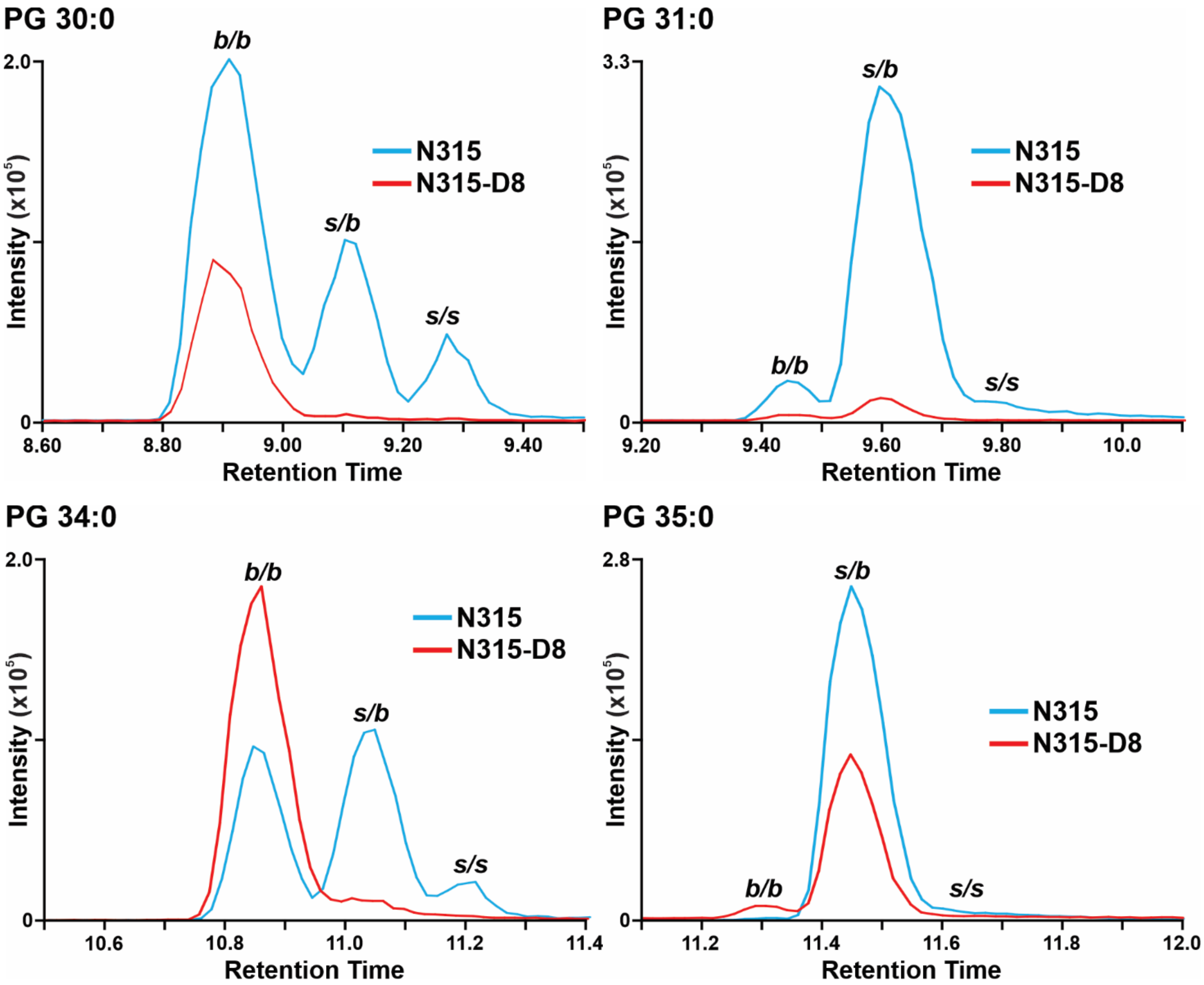

Figure S2. Extracted ion chromatograms for PGs in N315 and N315-D8.

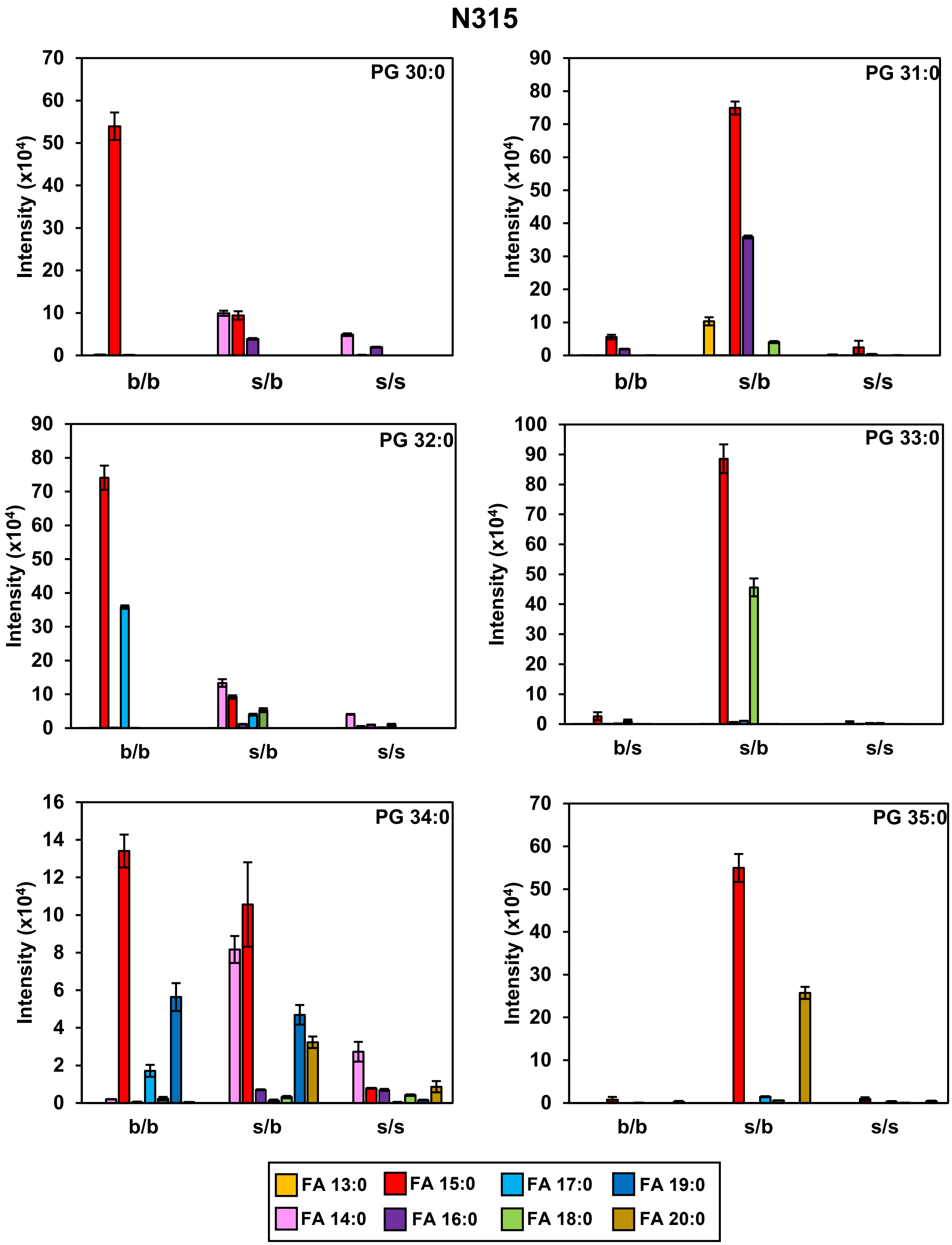

Figure S3. Fatty acyl tail composition of PGs in N315 determined by MS/MS.

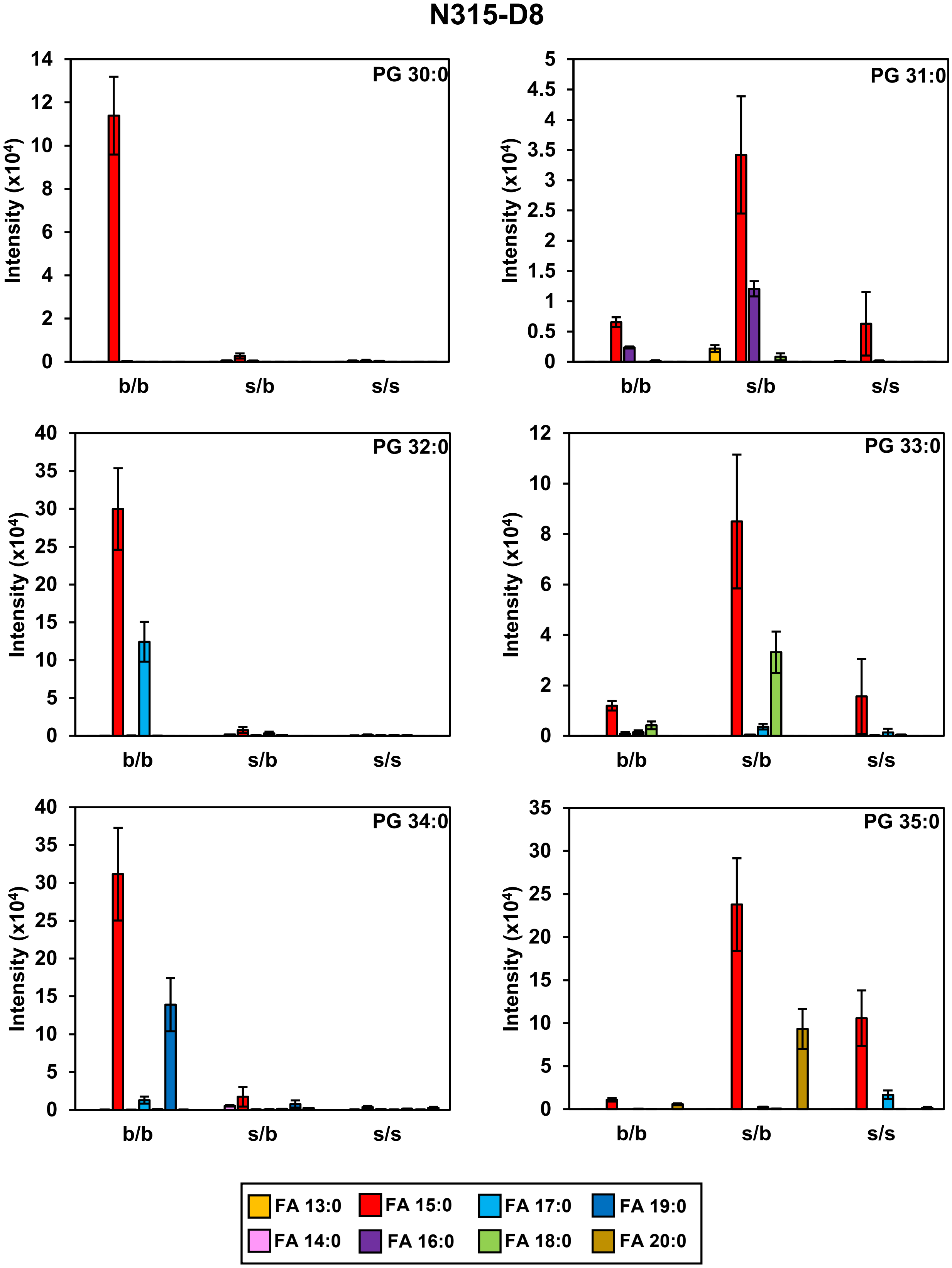

Figure S4. Fatty acyl tail composition of PGs in N315-D8 determined by MS/MS.

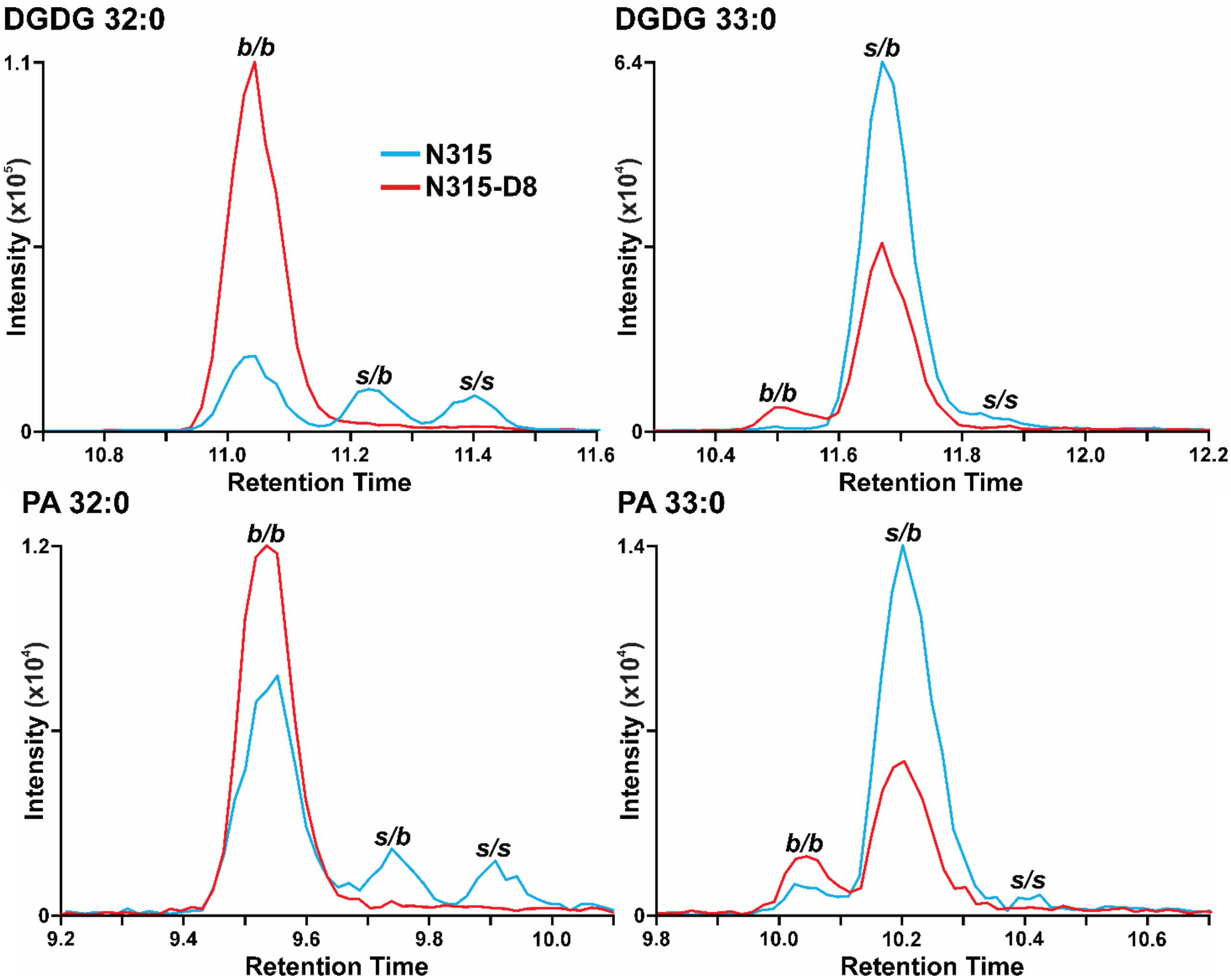

Figure S5. Extracted ion chromatograms of A) DGDG 32:0, B) DGDG 33:0, C) PA 32:0, and D) PA 33:0 for N315 (blue) and N315-D8 (red).

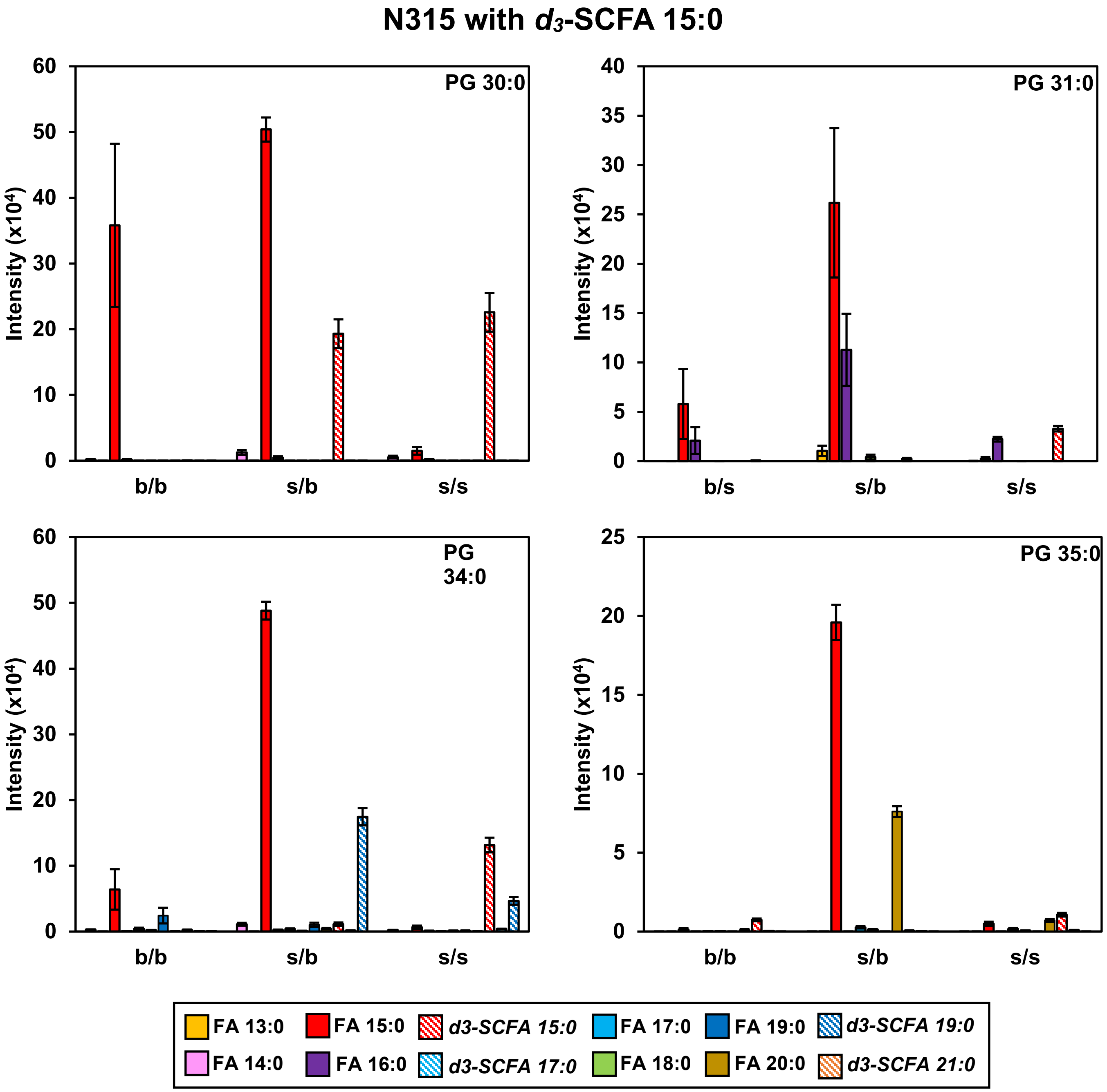

Figure S6. Fatty acyl tail compositions of PGs in N315 when grown in TSB containing d_3-SCFA 15:0.

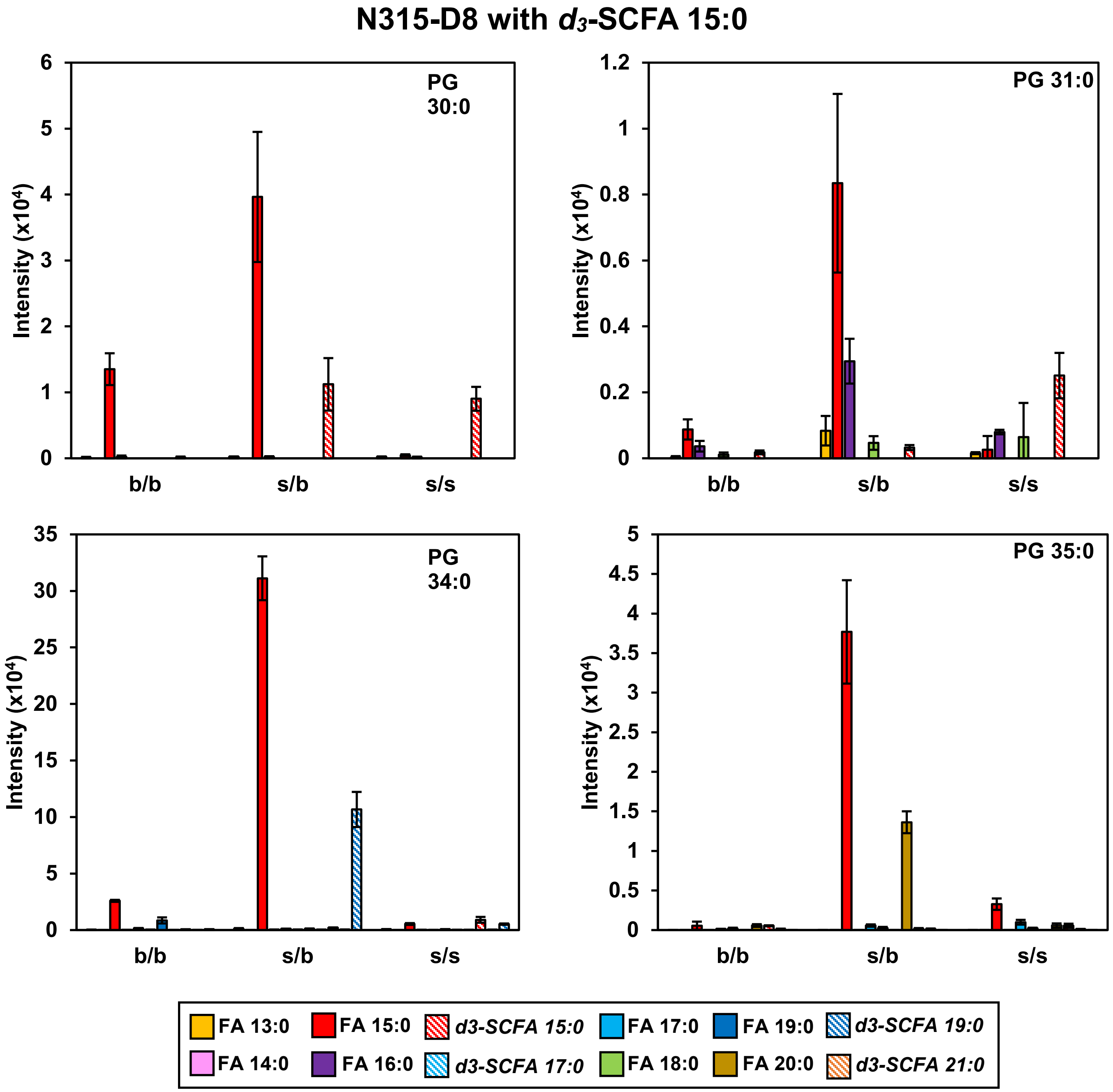

Figure S7. Fatty acyl tail compositions of PGs in N315-D8 when grown in TSB containing d_3-SCFA 15:0.

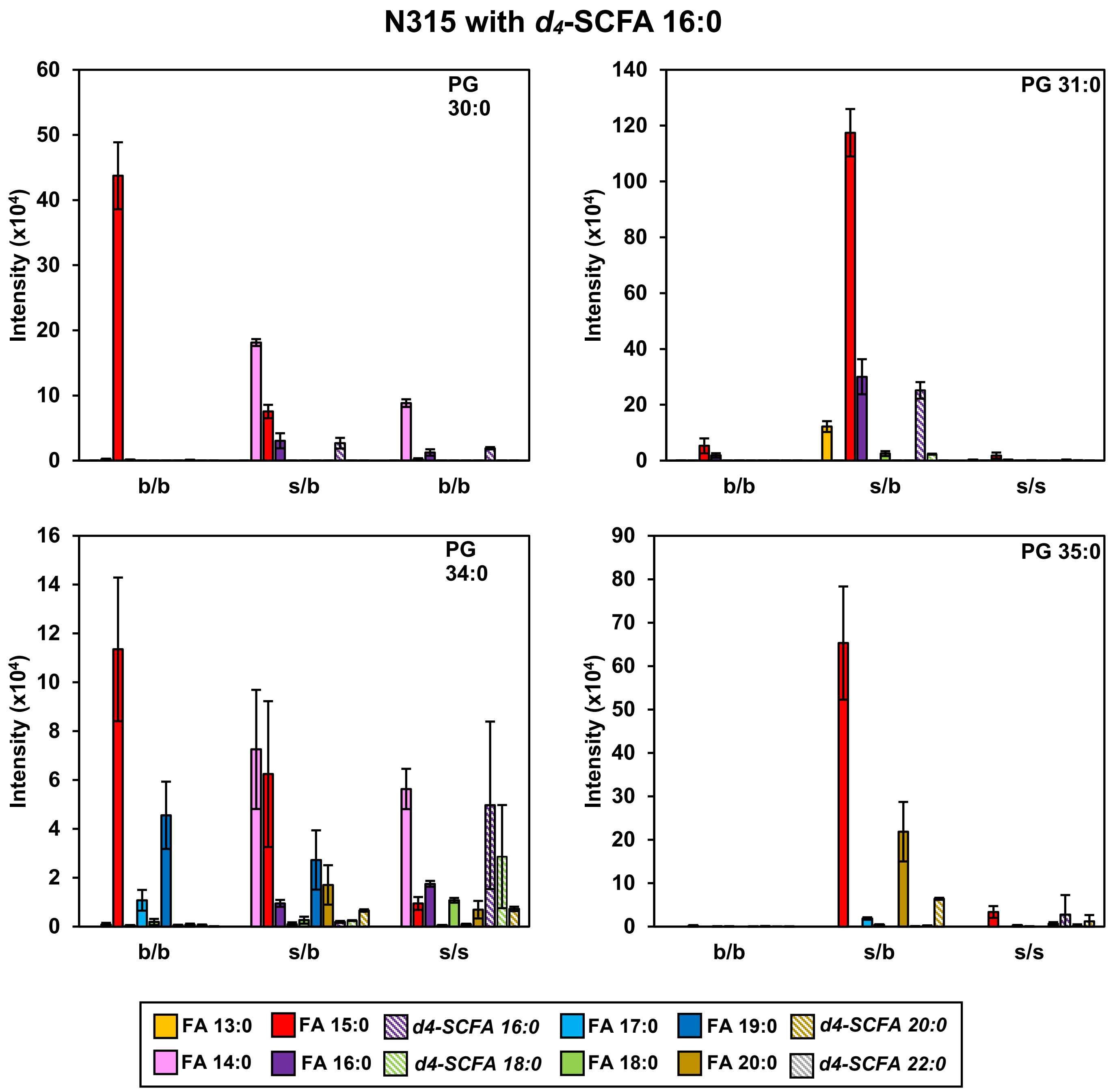

Figure S8. Fatty acyl tail compositions of PGs in N315 when grown in TSB supplemented with d_4-SCFA 16:0.

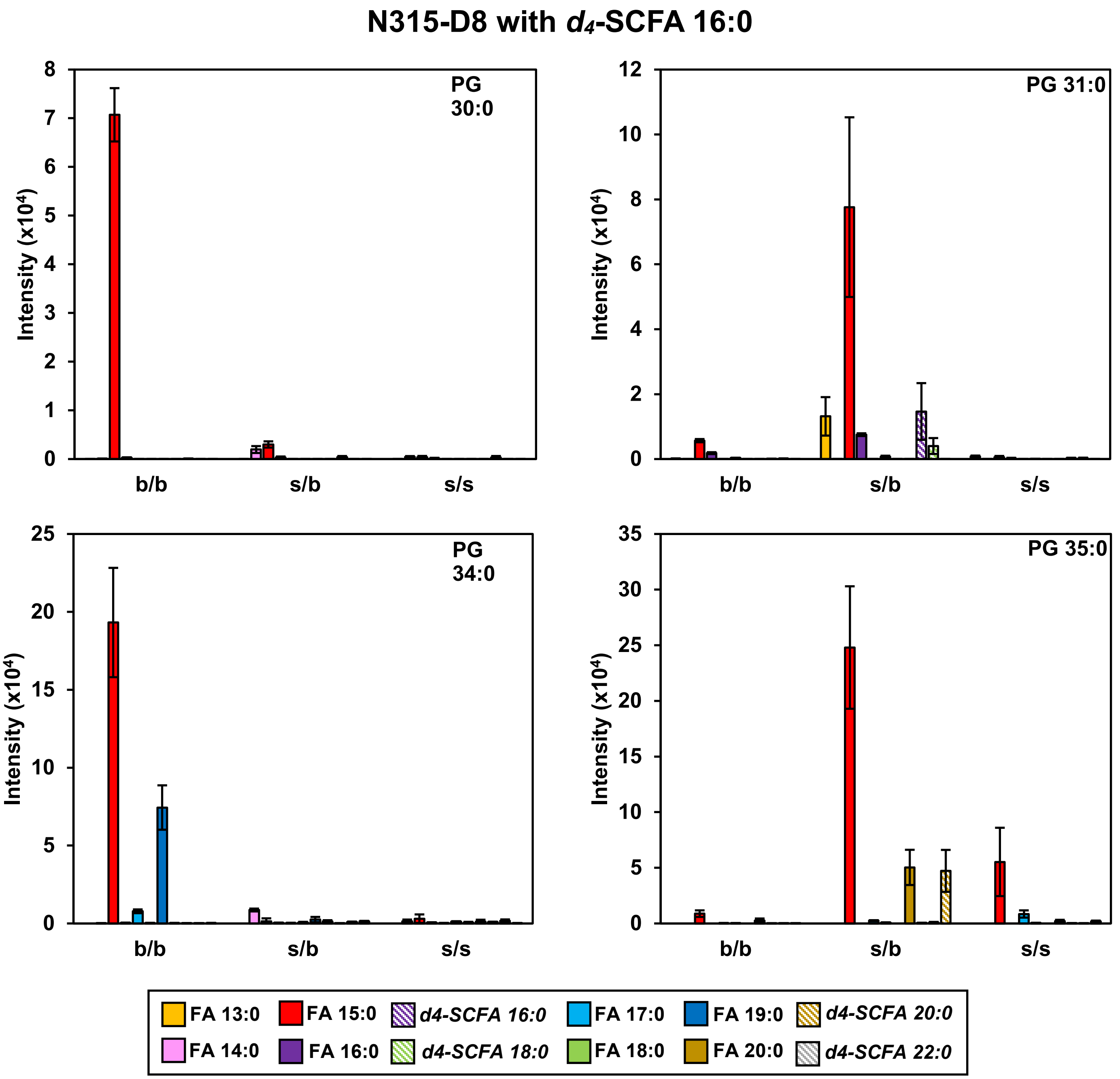

Figure S9. Fatty acyl tail compositions of PGs in N315-D8 when grown in TSB supplemented with d_4-SCFA 16:0.

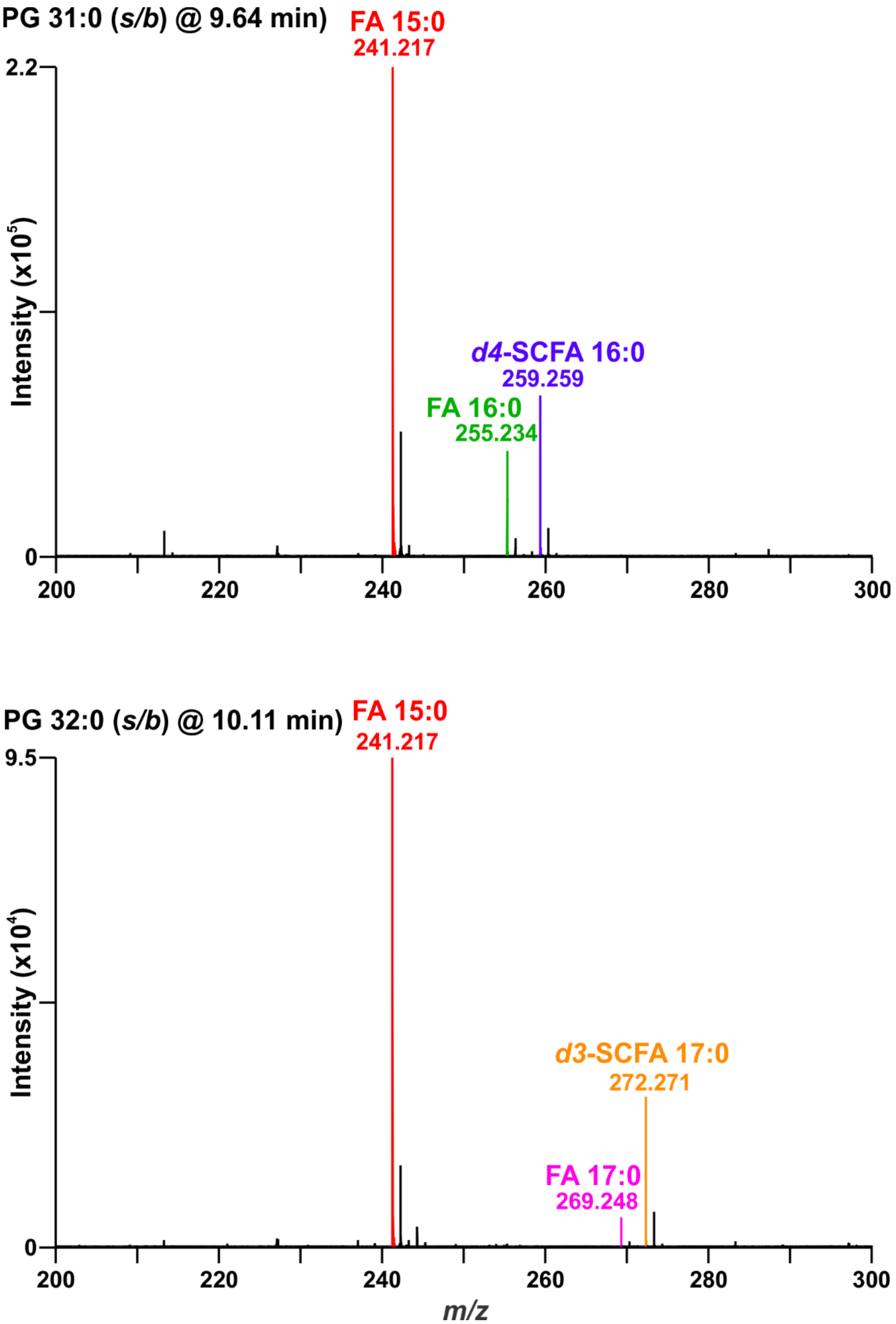

Figure S10. Fatty acyl tail composition of the *s/b* isomers of PG 31:0 and PG 32:0 in N315 after supplementation with d_4-SCFA 16:0 and d_3-SCFA 15:0 show that exogenous FAs incorporate at the *sn-1* position based on acyl tail fragment intensities.

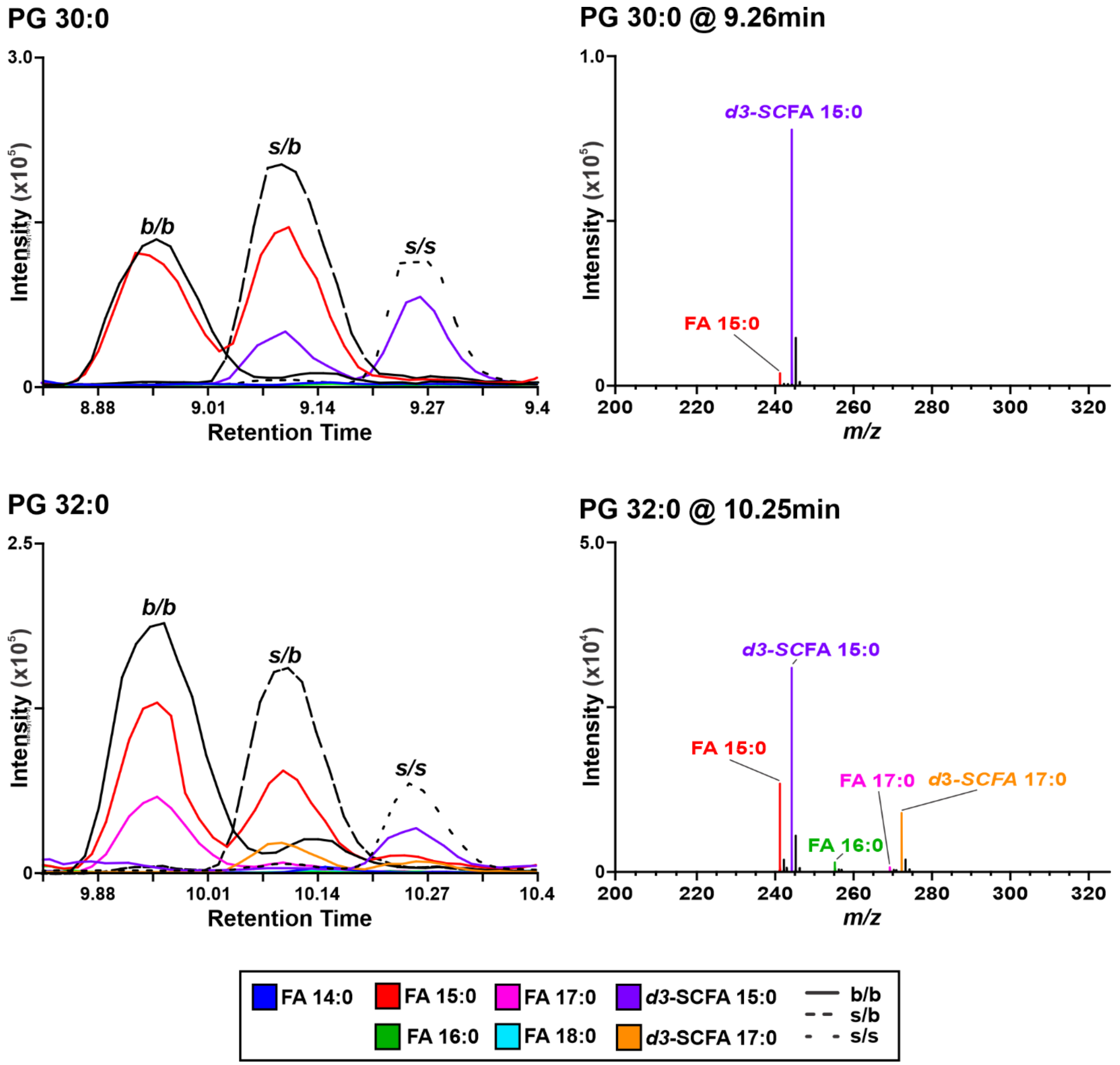

Figure S11. Fatty acyl tail composition of the *s/s* isomers of PG 30:0 and PG 32:0 in N315 after supplementation with d_3-SCFA 15:0 show the presence of PGs with fully-exogenous acyl tails.

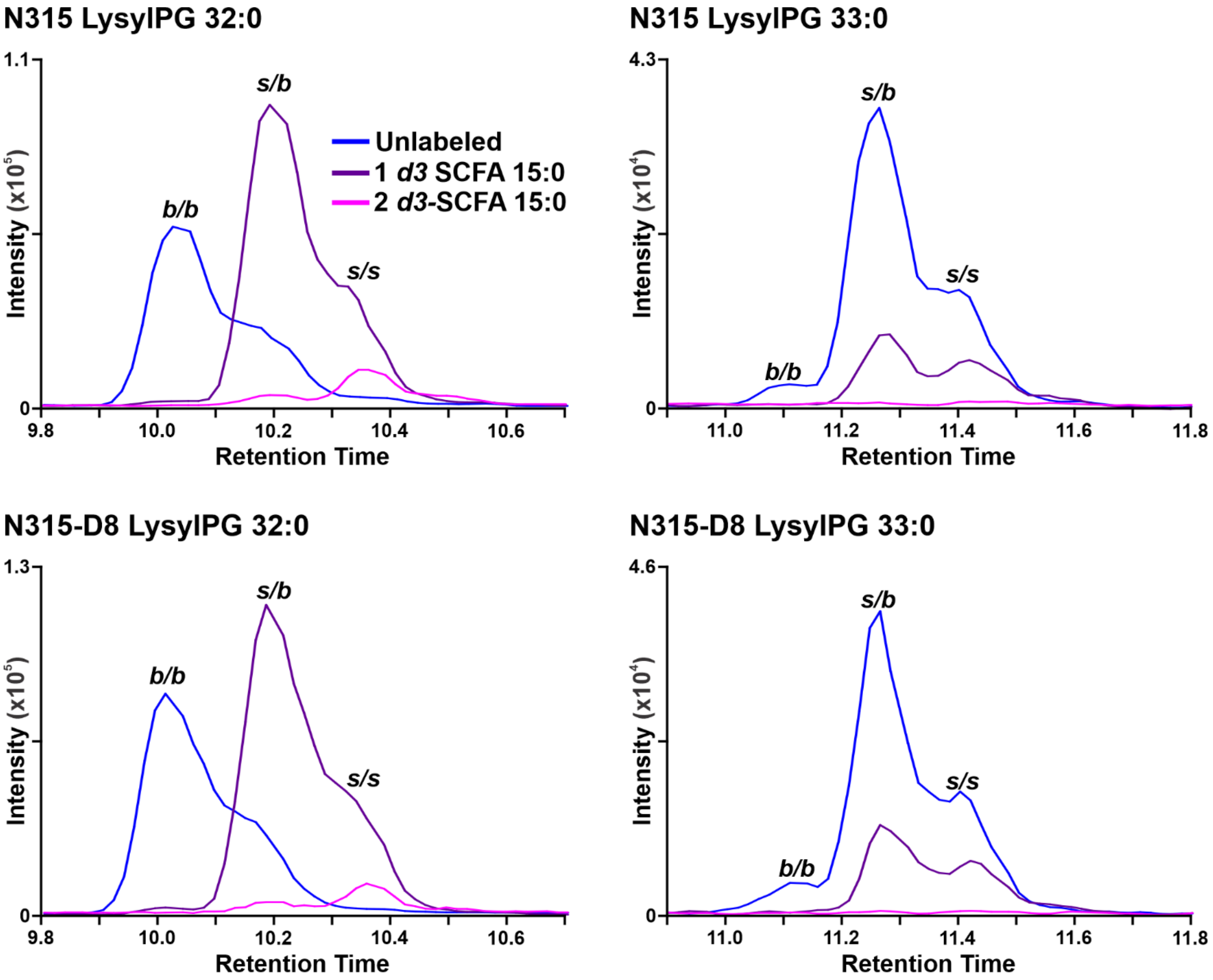

Figure S12. Extracted ion chromatograms of LysylPGs from N315 and N315-D8 grown in TSB supplemented with d_3-SCFA 15:0.

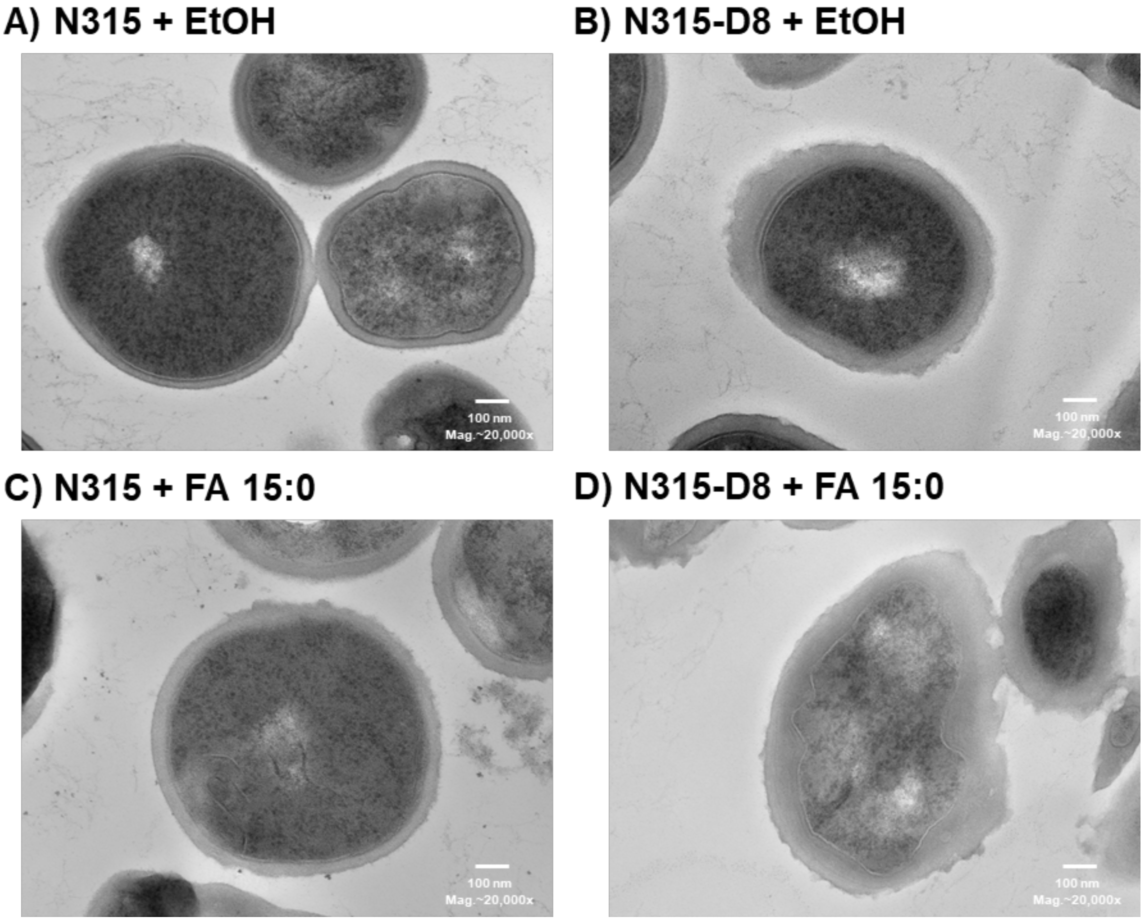

Figure S13. TEM images of N315 and N315-D8 cells that were treated with ethanol (as a vehicle control), or SCFA 15:0 in ethanol.

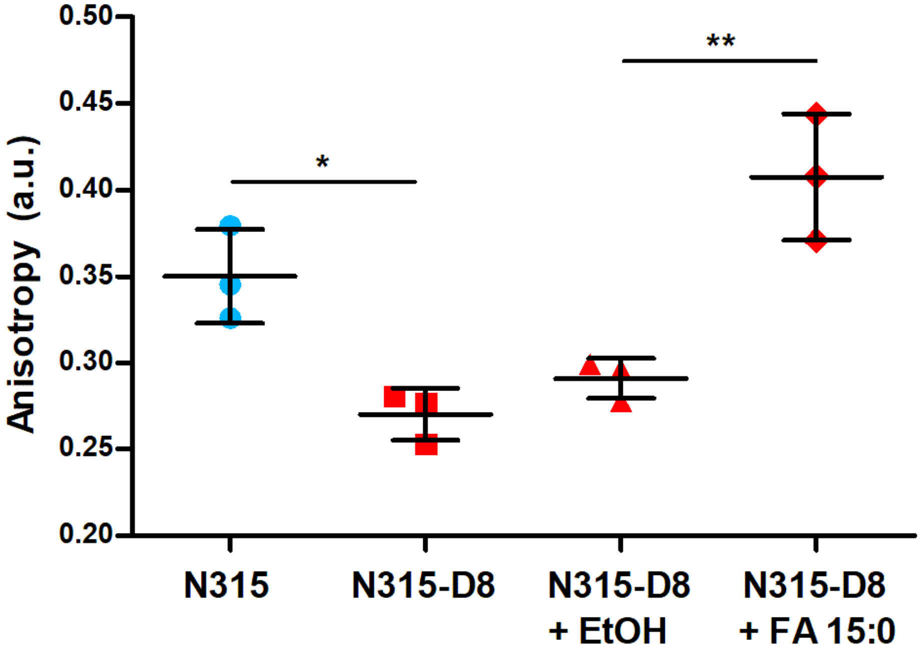

Figure S14. Anisotropic values of N315 and N315-D8 grown in TSB and N315-D8 supplemented with ethanol as a control and SCFA 15:0.

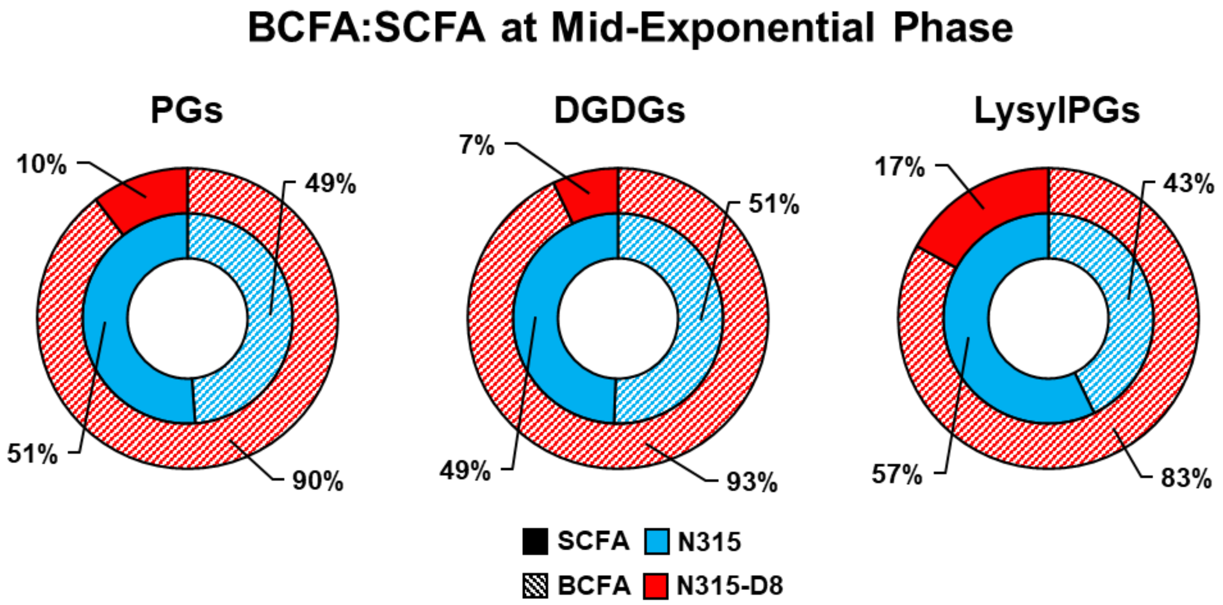

Figure S15. Distribution of BCFAs and SCFAs in PGs, DGDGs, and LysylPGs at mid-exponential phase in N315 and N315-D8.

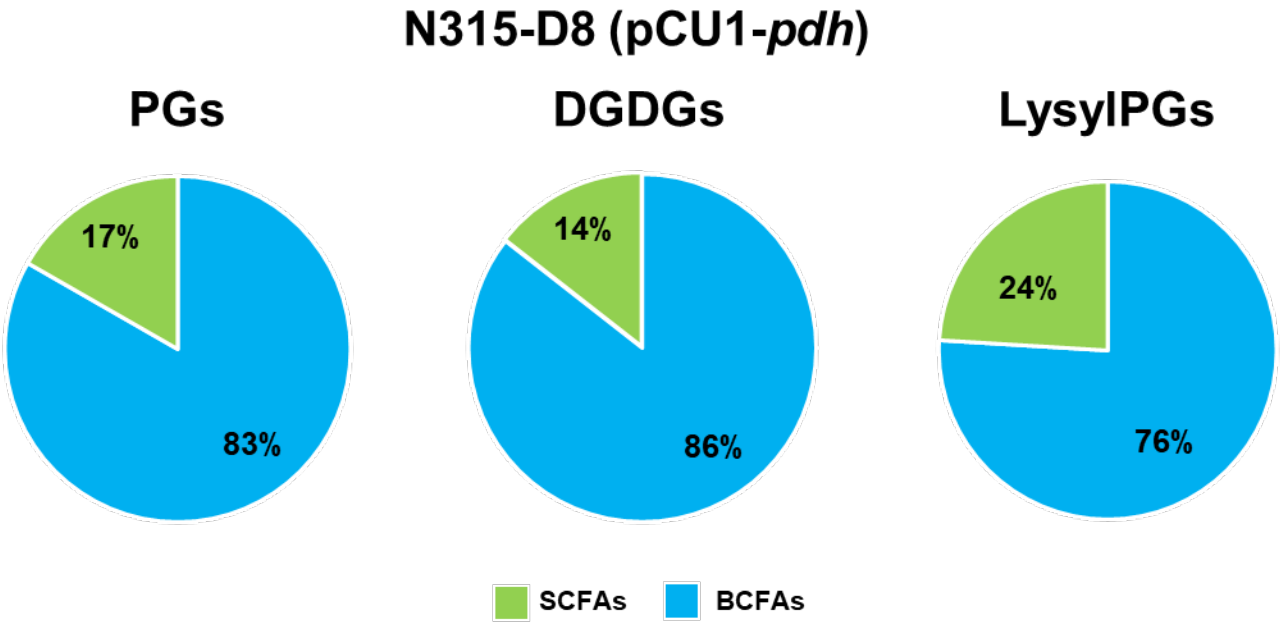

Figure S16. Pie charts of BCFA:SCFA for PGs, DGDGs, and LysylPGs in N315-D8 (pCU1-*pdh*).

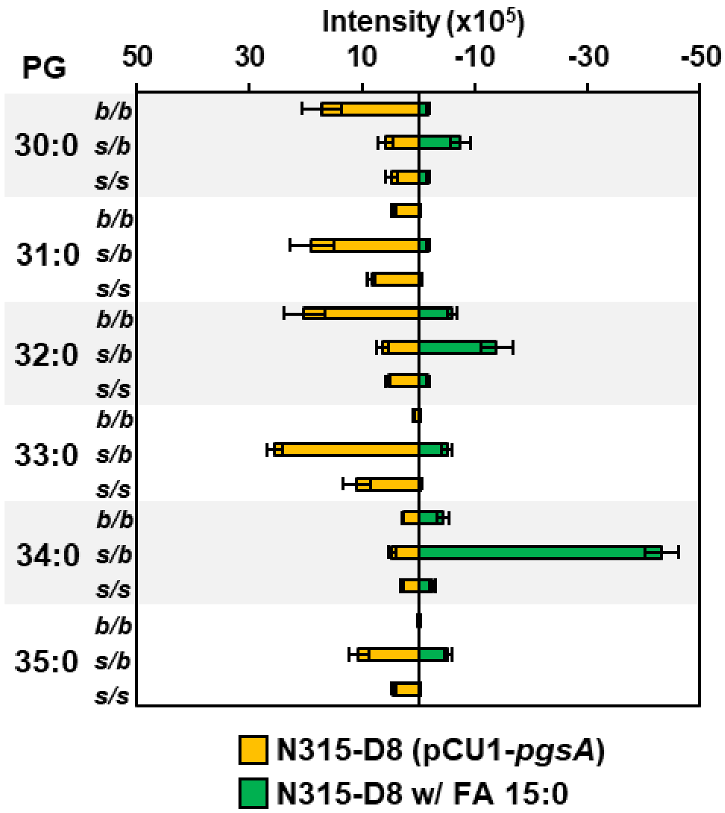

Figure S17. Distribution of BCFAs and SCFAs within PGs of N315-D8 (pCU1-*pgsA*) and N315-D8 grown in TSB supplemented with SCFA 15:0.

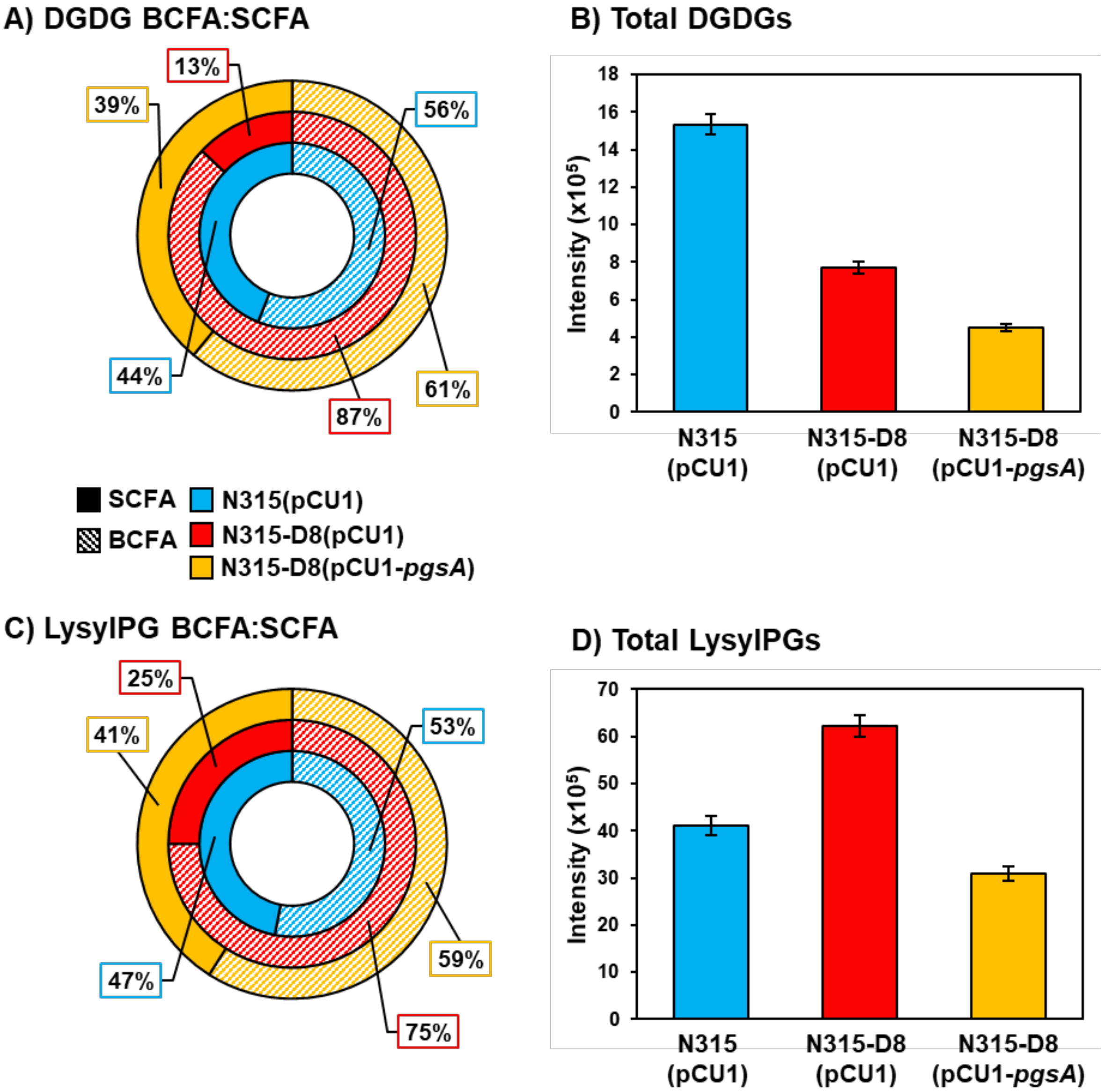

Figure S18. BCFA:SCFA ratios for A) DGDGs and C) LysylPGs, along with their total abundances (B,D), in N315 (pCU1), N315-D8 (pCU1) and N315-D8 (pCU1-*pgsA*).

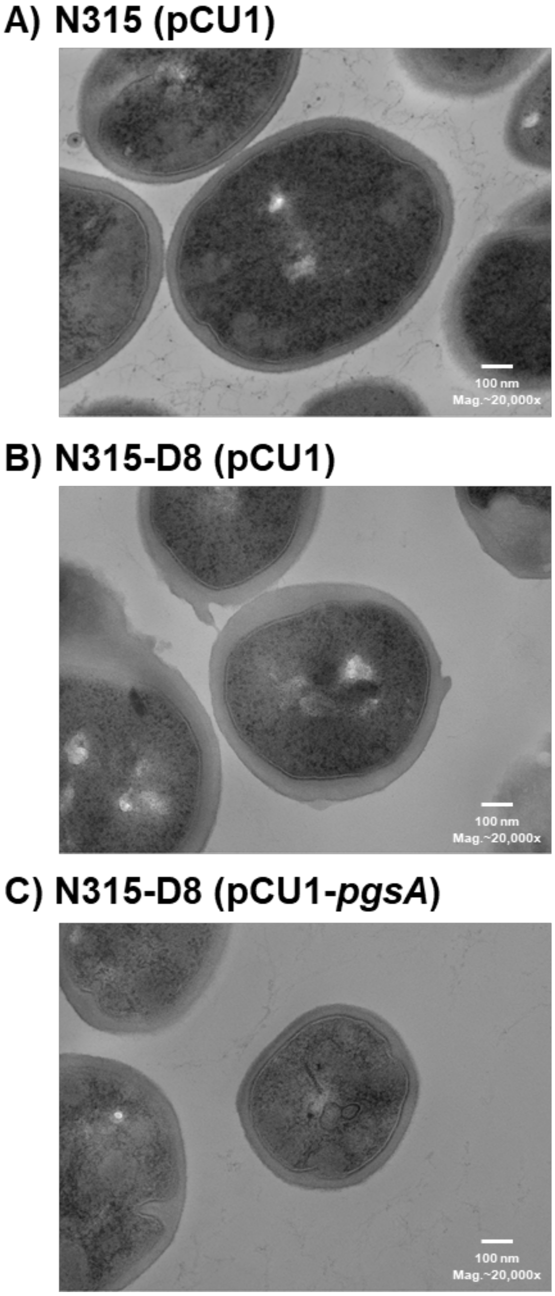

Figure S19. Representative TEM images of A) N315 (pCU1), B) N315-D8 (pCU1), and C) N315-D8 (pCU1-*pgsA*).

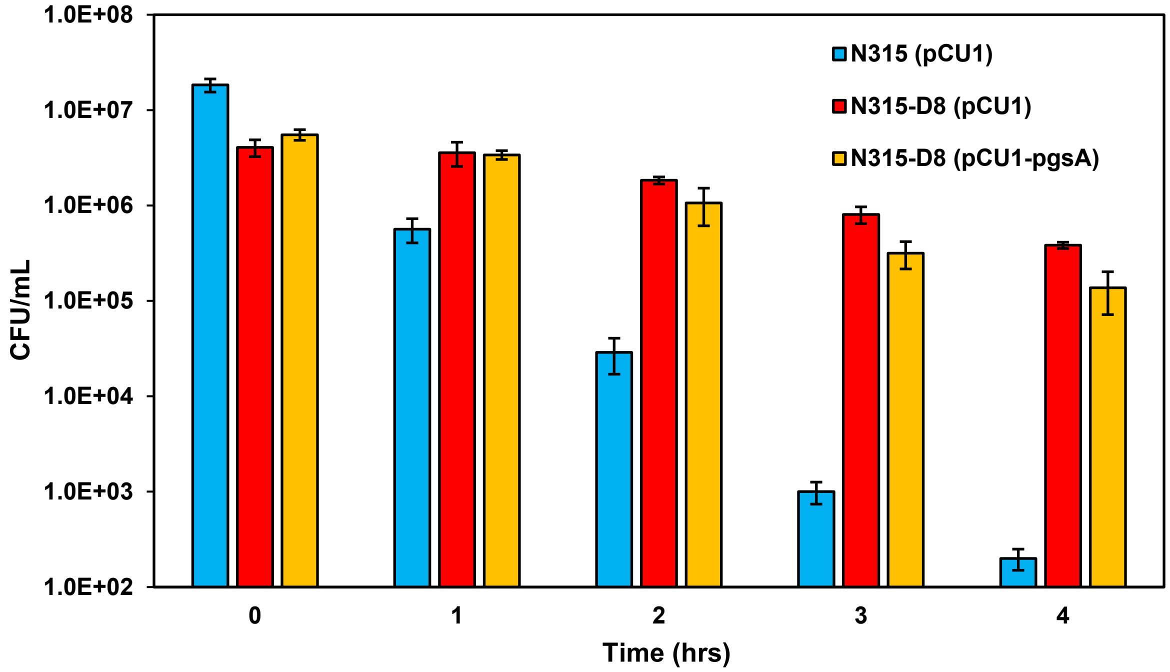

Figure S20. Number of cells (in CFU/mL) killed over 4 hours of daptomycin exposure (160 µg/mL in TSB) for N315 (pCU1), N315-D8 (pCU1) and N315-D8 (pCU1-*pgsA*).

Table S2. Daptomycin Minimum Inhibitory Concentrations.

**Strain**	**Daptomycin MIC* (µg/mL)**
N315	0.5
N315(pCU1)	0.5
N315-D8	8
N315-D8(pCU1)	8
N315-D8(pCU1-*pdh*)	8
N315-D8(pCU1-*pgsA*)	4

*Determined in Mueller-Hinton broth containing 30 mg/L $CaCl_2$.
